# Engineering of an Avidity-Optimized CD19-Specific Parallel Chimeric Antigen Receptor That Delivers Dual CD28 and 4-1BB Co-Stimulation

**DOI:** 10.3389/fimmu.2022.836549

**Published:** 2022-02-09

**Authors:** Leena Halim, Kushal K. Das, Daniel Larcombe-Young, Adam Ajina, Andrea Candelli, Reuben Benjamin, Richard Dillon, David M. Davies, John Maher

**Affiliations:** ^1^Chimeric Antigen Receptor (CAR) Mechanics Laboratory, Guy’s Cancer Centre, School of Cancer and Pharmaceutical Sciences, King’s College London, London, United Kingdom; ^2^Lumicks, Amsterdam, Netherlands; ^3^Faculty of Life Sciences and Medicine, School of Cancer and Pharmaceutical Sciences, King’s College London, London, United Kingdom; ^4^Department of Clinical Haematology, King’s College Hospital National Health Service (NHS) Foundation Trust, London, United Kingdom; ^5^Department of Clinical Haematology, Guy’s and St Thomas’ National Health Service (NHS) Foundation Trust, London, United Kingdom; ^6^Department of Medicine and Molecular Genetics, King’s College London, London, United Kingdom; ^7^Leucid Bio, Guy’s Hospital, London, United Kingdom; ^8^Department of Clinical Immunology and Allergy, King’s College Hospital National Health Service (NHS) Foundation Trust, London, United Kingdom; ^9^Department of Immunology, Eastbourne Hospital, Eastbourne, United Kingdom

**Keywords:** chimeric antigen receptor, avidity, CD19, parallel CAR, co-stimulation, CD28, 4-1BB

## Abstract

Co-stimulation is critical to the function of chimeric antigen receptor (CAR) T-cells. Previously, we demonstrated that dual co-stimulation can be effectively harnessed by a parallel (p)CAR architecture in which a CD28-containing second generation CAR is co-expressed with a 4-1BB containing chimeric co-stimulatory receptor (CCR). When compared to linear CARs, pCAR-engineered T-cells elicit superior anti-tumor activity in a range of pre-clinical models. Since CD19 is the best validated clinical target for cellular immunotherapy, we evaluated a panel of CD19-specific CAR and pCAR T-cells in this study. First, we generated a panel of single chain antibody fragments (scFvs) by alanine scanning mutagenesis of the CD19-specific FMC63 scFv (V_H_ domain) and these were incorporated into second generation CD28+CD3ζ CARs. The resulting panel of CAR T-cells demonstrated a broad range of CD19 binding ability and avidity for CD19-expressing tumor cells. Each scFv-modified CAR was then converted into a pCAR by co-expression of an FMC63 scFv-targeted CCR with a 4-1BB endodomain. When compared to second generation CARs that contained an unmodified or mutated FMC63 scFv, each pCAR demonstrated a significant enhancement of tumor re-stimulation potential and IL-2 release, reduced exhaustion marker expression and enhanced therapeutic efficacy in mice with established Nalm-6 leukemic xenografts. These data reinforce the evidence that the pCAR platform delivers enhanced anti-tumor activity through effective provision of dual co-stimulation. Greatest anti-tumor activity was noted for intermediate avidity CAR T-cells and derived pCARs, raising the possibility that effector to target cell avidity is an important determinant of efficacy.

## Introduction

Chimeric antigen receptors (CARs) are fusion molecules that re-direct lymphocyte specificity against cell surface targets. Immunotherapy with CD19-specific CAR T-cells has achieved dramatic impact in the treatment of relapsed/refractory B-cell leukemias and lymphomas. The key event that propelled this technology to become the largest growth area in immuno-oncology (1) was the inclusion of either CD28 or 4-1BB co-stimulatory elements within the CAR endodomain (2, 3). Evaluation of these so-called second generation (2G) CARs in human T-cells revealed that they confer a significantly enhanced capacity to mediate target-dependent proliferation and cytokine release, when compared to first generation (1G) receptors that only deliver an activating signal (4, 5). Currently, there are four licensed CD19-specific CAR T-cell products available worldwide, all of which contain an FMC63 single chain antibody fragment (scFv) and either a CD28 or 4-1BB co-stimulatory domain. Given the therapeutic failure of earlier designs, this highlights the vital importance of co-stimulation in this clinical breakthrough.

There is considerable evidence that combined provision of both CD28 and 4-1BB co-stimulation can synergistically enhance T-cell immune responses (6–8). Although these receptors activate overlapping signaling pathways, strength and kinetics of response differ markedly. While CD28-containing CARs elicit faster and larger scale signaling flux, 4-1BB favors a less intense but longer lasting response (9). We have previously shown that CAR T-cell co-stimulation both by CD28 and 4-1BB is optimally delivered by two separate fusion receptors. To leverage this, we engineered parallel (p)CARs in which a CD28-containing 2G CAR is co-expressed with a 4-1BB containing chimeric co-stimulatory receptor (CCR) (10). We observed that pCAR T-cells demonstrate more durable activity in several tumor models, indicated by enhanced proliferation, cytokine release and cytolytic function and lowered expression of exhaustion and senescence markers. As a result, superior *in vivo* anti-tumor activity was consistently achieved by pCAR T-cells compared to linear CAR T-cells in which either CD28 or 4-1BB (2G CAR) (4, 5) or both of these domains (third generation CAR) (11) had been included. Moreover, we also observed that pCAR T-cells outperformed the combination of a 1G CAR and a dual CD28 + 4-1BB CCR (12), potentially consistent with the importance of membrane proximity in effective co-stimulation. In mice in which pCAR T-cells achieved complete tumor rejection, enhanced functional persistence of these cells was confirmed by successful rejection of a delayed tumor rechallenge (10).

CD19 is the most strongly validated target antigen for CAR T-cell immunotherapy. Unprecedented efficacy has been reported by several groups using CD19-specific CAR T-cells to treat relapsed refractory B-cell malignancy (13). However, while CD19-specific CAR T-cells have proven clinically transformative, they are not uniformly successful in the induction of complete remission (CR) of B-cell malignancy. In lymphoma, CR rates range between 50-67%, of which 40-63% are durable to 12-29 months. In B-cell ALL, the CR rate is higher at 69-92%, but relapse occurs in 21-58% within 14 months (14). Moreover, greater than 50% of patients who receive CD19-specific CAR T-cell immunotherapy ultimately develop progressive disease (15). Relapse in which CD19 expression is maintained is the most common scenario - particularly with CD28-containing 2G CARs - and this has been linked to limited persistence of CAR T-cells (16–18). Given the need for more potent and durable CAR T-cell strategies to target CD19, we set out here to develop CD19-targeted pCARs in order to test their potential utility in models of B-cell malignancy. To test the importance of relative avidity of the CAR and CCR targeting moiety within the pCAR, we generated a panel of CARs with differing relative avidity for CD19 and co-expressed these with a CCR in which an unmodified FMC63 scFv was used to confer target specificity.

## Materials and Methods

### Cell Lines

Nalm-6 cells were a gift of Dr Robert Köchl, (the Francis Crick Institute, London, UK). LO68 were a gift from Prof T Sethi and Raji cells a gift from Dr Linda Barber (both King’s College London, London, UK). 293T cells were obtained from the European Collection of Authenticated Cell Cultures. Tumor cell lines were grown in R10 or D10 medium, respectively comprising RPMI or DMEM supplemented with 10% FBS and GlutaMax. 293VEC-RD114™ cells retroviral packaging cells were a gift of Dr Manuel Caruso (Biovec Pharma, Québec, Canada) and were maintained in D10. Cell lines were validated by STR typing and were routinely monitored for mycoplasma contamination.

### Human Samples

Blood samples from healthy male and females aged between 18-65 years were obtained from healthy volunteers with approval of a National Health Service Research Ethics Committee (reference 09/H0804/92 and 18/WS/0047).

### Retroviral Constructs

All recombinant DNA constructs were expressed using the SFG retroviral vector. Complementary DNA encoding for the FMC63 scFv was designed using published sequences (19) (GenBank HM852952.1) and synthesized by Integrated DNA Technologies (Coralville, IA, USA). The second-generation F-2 CAR was designed as follows; a CD8α leader was fused to the FMC63 scFv (arranged as variable light (V_L_)- variable heavy (V_H_) domains separated by a [SerGly4]_3_ linker). The synthetic cDNA was flanked with a 5’ Nco1 restriction site (that coincides with the start codon) and a 3’ Not1 restriction site. This fragment was cloned into the unique Nco1 and Not1 restriction sites of SFG A20-28z (20), placing the scFv cDNA upstream of the fused MYC epitope tag_CD28 hinge/transmembrane/endodomain_CD3ζ endodomain.

The complementarity determining region (CDR)3 of the V_H_ domain within the FMC63 scFv was identified using Abysis.^1^ To generate CAR variants with an altered ability to bind CD19, an alanine (A) residue was substituted for the first or second glycine (G01, G02) or alternatively for the third, fourth or fifth tyrosine (Y03-Y05) within the CDR3 region of the V_H_ domain. These substitutions were introduced into the F-2 CAR *via* single site mutagenesis (Genscript, Piscataway, NY, USA).

Codon optimized cDNAs encoding candidate pCARs were synthesized by Genscript (Piscataway, NJ, USA). To engineer pCARs, an Nco1 flanked CCR cDNA was engineered that comprises a linear fusion of the following elements: a colony-stimulating factor-1 receptor leader peptide, FMC63 scFv binding domain (V_L_-V_H_ order), CD8α spacer and transmembrane domain (codons 137-208), a 4-1BB co-stimulatory endodomain (codons 214-255), C-terminal FLAG epitope tag, an RRKR furin cleavage site, SGSG linker and *Porcine Teschovirus* (P2A) ribosomal skip peptide. This cDNA fragment was inserted into the unique Nco1 restriction site containing the start codon of each the V_H_ CDR3-mutated CARs within SFG, thereby placing the CCR cDNA upstream of the CAR cDNA. Codon wobbling was used to minimize direct repeats within these vector inserts.

A codon optimized cDNA encoding for human CD19 was synthesized by Genscript and cloned into the Nco1 site of SFG. The SFG ffLuc/RFP vector which encodes both firefly luciferase and dsTomato red fluorescent protein has been described previously (20).

### Transduction and Expansion of Human T-Cells

Viral vector was prepared as described using 293VEC-RD114™ cells (21) or by triple transfection of 293T cells. In brief, 1.65x10^6^ low passage 293T cells in 11mL IMDM + 10% FBS were evenly distributed in a 10cm plate. After 8-24h, GeneJuice (30µL; Sigma-Aldrich, Poole UK; Cat# 70967) was added to 470µL IMDM (no serum) and mixed gently. After incubation for 5 minutes at room temperature, 3.125µg RD114 plasmid (a gift of Prof M Collins, University College London, London UK), 4.6875µg pEQ-Pam3 plasmid (a gift of Dr M Pulé, University College London, London UK) and 4.6875µg SFG vector of interest were added to the GeneJuice/medium mixture, mixed gently and incubated for 15 minutes at room temperature. The transfection mixture was dropwise to the plate and gently swirled to ensure even distribution. After incubation for 48h at 37°C, 5% CO_2,_ medium was removed for snap freezing using an ethanol dry ice bath and replaced. After a further 24h, this procedure was repeated. Frozen virus was stored in aliquots at -80°C. Retroviral transduction and culture of phytohemagglutinin- or CD3+CD28 Dynabead-activated T-cells using RetroNectin (Takara, Orchard Parkway, San Jose, CA, USA; Cat# T100B)-coated plasticware was performed as described (4, 22).

### Flow Cytometry Analysis

All cell surface antigen staining reactions were performed for 30 min on ice. CAR expression was detected with mouse 9e10 hybridoma supernatant (20µL per test, produced in house) followed by goat anti-mouse IgG/A/M-RPE (Dako/Agilent, Santa Clara CA, USA; Cat# R048001-2) or Alexa Fluor^®^ 647 goat anti-mouse/human IgG (Jackson ImmunoResearch, Europe Ltd, Ely, UK; Cat# 115-605-003; RRID: AB_2338902). CD19 expression was detected using FITC anti-human CD19 (Biolegend, San Diego, CA, USA; Cat# 302205; RRID AB_314235). Cell surface exhaustion markers were detected using PE-anti-human CD279 (PD1, Biolegend; Cat# 621607; RRID AB_2832827), APC-anti-human CD366 (TIM3, Biolegend; Cat# 345011; RRID AB_2561717) and Alexa Fluor^®^ 647 anti-human CD233 (LAG3, Biolegend Cat# 369303; RRID AB_2566479).

Intracellular staining was performed by fixation with 0.4% formaldehyde followed by permeabilization using PBS + 0.5% BSA + 0.1% saponin. Cells were subsequently stained for 30 min on ice. CCR expression was detected using APC-conjugated anti-DYKDDDDL (Biolegend, Cat# 637307; RRID AB_2561496).

CD19-Fc binding studies were performed by addition of 0.5µg/mL or 1.0µg/mL CD19-Fc (contains human IgG1 Fc; Acro Biosystems, Newark, DE, USA; Cat# CD9-H5251) to 0.5 x 10^6^ cells for 30 minutes on ice. Bound protein was detected with Alexa-Fluor^®^ 647 conjugated anti-human IgG (Jackson ImmunoResearch, Europe Ltd, Cat# 109-605-006; RRID: AB_2337881). Since the scFv used in all CARs is of murine origin, this reagent does not bind directly to any CAR. To normalize binding to transduction efficiency (as determined by staining with anti-MYC antibody), the following formula was used.

% Normalized binding = % CD19-Fc binding to CAR T-cells - % CD19-Fc binding to untransduced T-cells/% transduced (MYC^+^) cells x 100.

All gates were set using isotype control antibodies or fluorescence minus one controls. Where necessary, a viability stain (Zombie NIR™ Fixable Viability Kit, Biolegend; Cat# 423105) was included and non-specific binding of the antibodies was limited by using an appropriate Fc blocking reagent prior to the staining steps.

All flow cytometry was performed using a FACSCalibur cytometer with CellQuest Pro software or BD LSRFortessa cytometer with BD FACSDiva software and data was analyzed using FlowJo, LLC.

### Enzyme-Linked Immunosorbent Assay

Supernatants collected from co-culture of tumor cells with CAR T-cells were analyzed using a human interferon (IFN)-γ (Cat# 88-7316-76, RRID : AB_2575072) or human interleukin (IL)-2 (Cat# 88-7025-76, RRID : AB_2574956) enzyme-linked immunosorbent assay (ELISA) as described by the manufacturers (ThermoFisher Scientific, Horsham, UK). In pooled re-stimulation assays, cytokine production was set to zero in each cycle after T-cell cultures failed.

### Cytotoxicity Assays

Tumor cells were incubated with T-cells at specified effector to target (E:T) ratios. In the case of adherent targets, residual tumor cell viability was quantified using an MTT assay at the indicated time point. After removal of the supernatant and residual T-cells, MTT (Apollo Scientific; Cat# BID2165) was added at 500 µg/mL in D10 medium for 40 minutes at 37°C and 5% CO_2_. Formazan crystals were resuspended in DMSO and absorbance was measured at 560 nm. Alternatively, tumor cell viability was monitored by luciferase assays. D-luciferin (R&D Systems (Biotechne), Cat# 122799) was added at 150 mg/mL immediately prior to luminescence reading. In each case, tumor cell viability was calculated using the following formula:

Absorbance or Luminescence of tumor cells cultured with T-cells/Absorbance or Luminescence of untreated monolayer alone x 100%.

### Tumor Re-Stimulation Assays

CD19-expressing LO68 target cells and CAR/pCAR T-cells were co-cultured in triplicates in a 24-well plate at a 1:1 E:T ratio (1 x 10^5^ cells each) without further addition of cytokines. Supernatant was harvested after 24h for cytokine analysis. At 72 hours tumor cell viability was assessed by MTT assay. If T-cells achieved more than 60% tumor cell destruction, they were restimulated on a fresh tumor monolayer. This process was repeated until T-cells failed to destroy >60% of tumor cell monolayers.

### CAR Binding Studies – z-Movi

CD19-engineered LO68 tumor cells were seeded in a z-Movi microfluidic chip (Lumicks, Amsterdam, Netherlands) coated with poly-L-lysine and cultured for 16 hours. The next day, flow sorted CAR-T cells were serially flowed in the chips and incubated with the target cells for 5 minutes prior to initializing a 3-minute linear force ramp. During the force ramp, the z-Movi device (Lumicks) captures a time series of images using a bright field microscope integrated into the platform. Detached cells were levitated towards the acoustic nodes, allowing the tracking of cells based on their XY positions. Changes in the Z-position results in a change in the diffraction pattern, which allows the distinction between cells adhered to the substrate and cells suspended to the acoustic nodes. This information is used to correlate cell detachment events with a specific rupture force. Cell detachment was acquired using z-Movi Tracking_v1.6.0 and post experiment image analysis was done using Cell Tracking offline analysis_v2.1. Data are presented as median acoustic force (rForce) which is the relative force required to elicit cell detachment and calibrated to 10µM polystyrene beads. Avidity score is calculated by the software as the ratio of the mean relative force (rForce) required to detach the CAR T-cells from an LO68-CD19^+^ tumor monolayer, when compared to untransduced controls. Thus, untransduced cells in each run have an avidity score of 1.

### Evaluation of CAR T-Cell Number *In Vivo*

After sacrifice, single cell suspensions were made of spleen by maceration through a cell strainer. Blood was collected by cardiac puncture from euthanised mice into Eppendorf tubes containing citrate-dextrose (Sigma) as anticoagulant. Red blood cells were lysed using 1X RBC Lysis Buffer (Biolegend) according to manufacturer’s instructions. Cells were pelleted by centrifugation at 300g for 5 minutes, resuspended in 1mL and then stained with CountbrightTM beads (Cat# 36950, Thermofisher), according to the manufacturers’ instructions.

### *In Vivo* Xenograft Studies

All *in vivo* experimentation adhered to U.K. Home Office guidelines, as specified in project licence number 70/7794 or P23115EBF and was approved by the King’s College London animal welfare and ethical review body (AWERB). NOD SCID γc null (NSG) mice were used for *in vivo* studies and were purchased from Charles River Laboratories (Harlow, UK). Mice were 6-10 weeks old when used for experiments. Similar numbers of male and female mice were used throughout. Nalm-6 tumor cells were transduced with SFG ffLuc/RFP and were purified by flow sorting prior to engraftment *in vivo* by i.v. injection of 5 x 10^5^ cells. Mice were allocated to experimental groups based on similar average tumor burden prior to treatment. Five days after Nalm-6 injection, CAR/pCAR T-cells were administered i.v. at a dose of 5 x 10^5^ cells. To monitor tumor status, bioluminescence imaging (BLI) was performed using an IVIS Spectrum Imaging platform (PerkinElmer, Waltham, MA, USA) with Living Image software. Mice were injected i.p. with D-luciferin [150 mg/kg; R&D Systems (Biotechne)] and imaged under isoflurane anesthesia after 20 min. In all experiments, animals were inspected daily and weighed weekly.

### Statistical Analysis

All data are derived from biological replicates involving independent donors unless otherwise indicated. For analysis of multiple groups, statistical analysis was performed using one-way or two-way ANOVA test (depending on the number of independent variables) followed by Tukey’s multiple comparisons test. For non-parametrically distributed data, a Kruskal Wallis test was performed. Survival data were analyzed using a Log rank (Mantel-Cox) test. When only 2 groups were compared, a Student’s *t*-test or Mann-Whitney test was performed, depending on normality of the data. Correlation testing was performed using a Spearman test. All statistical analyses were performed using GraphPad Prism version 9.1.

## Results

### Generation of a Panel of FMC63 Modified CARs With Specificity for CD19

**Figure 1:**
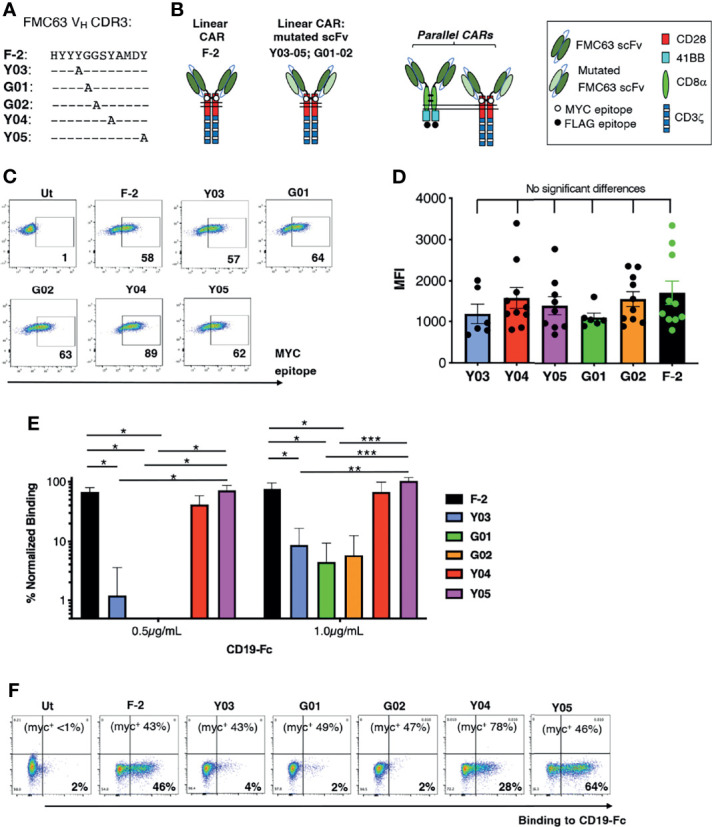
Engineering of a panel of CD19-specific CARs. **(A)** The indicated mutations were introduced into the V_H_ CDR3 region of an FMC63 scFv. **(B)** Unmodified and mutated FMC63 scFvs were used to target 2G (CD28+CD3ζ) CARs. The CAR containing the unmodified scFv was dubbed F-2 while CARs containing a modified scFv are named according to the amino acid within V_H_ CDR3 that has been substituted with alanine. Structure of CD19 pCARs is also shown. Each consists of a CD28+CD3ζ 2G CAR targeted by a mutated FMC63 scFv and co-expressed with a 4-1BB CCR in which an unmodified FMC63 scFv confers CD19 specificity. **(C)** Primary human T-cells were transduced with the indicated CD19-specific CARs and were analyzed by flow cytometry 5 days after gene transfer. Cells were incubated with 9e10 antibody, which detects an embedded MYC epitope tag within the CAR, followed by goat anti-mouse secondary antibody. Data are representative of 7 independent replicate experiments. **(D)** Mean fluorescence intensity of CAR expression (mean ± SEM, n=6-10). Statistical analysis was performed using one-way ANOVA. **(E)** CAR T-cells were incubated with CD19-Fc protein (0.5 or 1.0µg/mL). Binding of Fc fusion protein was detected with Alexa-Fluor^®^ 647 V_H_ conjugated anti-human IgG. The indicated proportion of CAR-expressing T-cells in each culture was determined by flow cytometry as described in C (mean +/- SEM, n=3 independent donor replicates). Following subtraction for non-specific binding to untransduced cells, data were normalized for the % CAR-expressing T-cells present. Statistical analysis was performed using one-way ANOVA. ***p < 0.001; **p < 0.01; *p < 0.05. One representative example of this analysis is also presented **(F)**. Cell surface CAR expression was detected using anti-MYC antibody (percentage indicated in the top of each panel). Binding to a CD19-Fc fusion protein is shown for each CAR T-cell population.

We focused on CARs that contain a CD28+CD3ζ endodomain since CD19 expression is most commonly retained at the time of disease relapse, following infusion of these cells (16–18). To generate a panel of scFvs with varying ability to bind CD19, CDR3 of the V_H_ domain within the CD19-specific FMC63 scFv was subjected to alanine scanning mutagenesis (**Figure 1A**). Chimeric antigen receptors were generated using an unmodified FMC63 scFv, dubbed F-2, in addition to these mutated scFvs, which were named according to mutation site within V_H_ CDR3 (e.g. Y03, G01, G02, Y04 or Y05; **Figures 1A, B**) (23). Following retroviral transduction, cell surface expression of all CARs could be demonstrated in human T-cells by flow cytometry, taking advantage of an embedded MYC epitope tag within the CAR spacer domain. Despite similar cell surface expression levels (allowing for donor to donor variability; **Figures 1C, D**), the resulting panel of CARs presented a broad range of CD19-binding activity (**Figure 1E**). A representative example of this analysis is shown in **Figure 1F**. Cell surface CAR expression is specified in the upper section of each panel, as detected using anti-MYC antibody. Panels show the binding of a CD19-IgG1 Fc fusion protein to each of these T-cell populations, which was detected using Alexa Fluor 647^®^-conjugated anti-human IgG.

### Avidity Analysis of CD19-Specific CAR T-Cells

**Figure 2:**
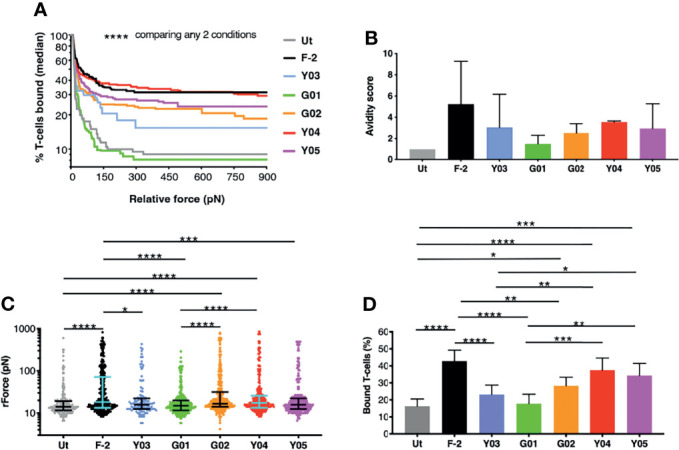
z-Movi analysis of CAR T-cell avidity for CD19^+^ LO68 tumor cells. **(A)** T-cells were engineered to express the F-2 (CD28+CD3ζ) CAR or 2G CAR derivates that contain a mutation in V_H_ CDR3 region. After flow sorting to purity, CAR T-cells were incubated on CD19^+^ LO68 tumor cells within a z-Movi microfluidic chip. Increasing acoustic force was applied and the median percentage of bound T-cells was determined over time. Statistical analysis was performed using a Kruskal-Wallis test, comparing % T-cells bound across the entire force ramp. **(B)** Avidity score represents the ratio of the mean relative force (rForce) per cell required to detach T-cells from the target CD19^+^ LO68 tumor monolayer, compared to untransduced (Ut.) T-cells (median + interquartile range, n=3-4 repeats from 2 independent donors). **(C)** Dot plot represents the rForce per cell required for detachment from the target cell monolayer. For clarity, a single representative run for one healthy donor is plotted in which each dot represents a single cell. Bars indicate median and interquartile range. Collectively, these dots generate the avidity curve shown in **(A)**, meaning that all avidity curves are built with >1000 single cell observations per donor. Statistical analysis was performed using a Kruskal-Wallis test. **(D)** Bar plot depicting the percentage of T-cells bound to the target cell monolayer after applying a minimal rForce (210 pN) required to detach a median of 90% untransduced (Ut.) T-cells (mean ± SEM of n=11 analyses incorporating 3 different healthy donors). Statistical analysis was performed using two-way ANOVA. ****p < 0.0001; ***p < 0.001; **p < 0.01; *p < 0.05.

We next ranked the avidity of these 2G CAR T-cells for CD19-expressing tumor cells using z-Movi analysis (24). CAR T-cells were purified by flow sorting and then serially flowed on LO68-CD19^+^ tumor cells (**Figure S1**) that had been pre-immobilized on microfluidic chips. Co-cultures were established for 5 minutes prior to the application of an acoustic force ramp to induce CAR T-cell detachment from the target cells. **Figure 2A** demonstrates the median percentage of T-cells that remained bound to the CD19^+^ monolayer over the course of acoustic force ramp application. Curves were compiled from 11 separate runs using T-cells from 3 independent donors in which at least 1000 single cell observations were collected per run. The data demonstrate that these T-cells display a spectrum of avidities for CD19-expressing target cells, with highly significant differences between all CARs tested. In **Figure 2B**, an avidity score has been calculated as the ratio of the mean relative force (rForce) required to detach the CAR T-cells from an LO68-CD19^+^ tumor monolayer, when compared to untransduced controls. Please note that only 3-4 technical replicates from 2 donors are included in this analysis, which may explain some differences relative avidity compared to data shown in **Figure 2A** (11 technical replicates from 3 donors). **Figure 2C** depicts a single representative run and illustrates the rForce required to detach individual CAR T-cells (each represented by a dot) during the application of the acoustic force ramp. The percentage of CAR T-cells that remain bound to the monolayer upon application of the minimal rForce required to dislodge a median of 90% untransduced cells is depicted in **Figure 2D**. Minimal rForce values for each CAR T-cell population correlated with % normalized binding of these cells to CD19-Fc (Pearson *r* = 0.85 (*p* = 0.015) and *r* = 0.83 (*p* = 0.021) at 0.5µg/mL and 1.0µg/mL respectively). Collectively, these data show that Y03 and G01-expressing CAR T-cells exhibited markedly reduced target cell avidity compared to F-2-expressing cells, close to (Y03) or comparable (G01) to that of untransduced T-cells. By contrast, the avidity of Y04-expressing CAR T-cells was similar to F-2 cells, while intermediate avidity was seen with Y05 and G02 CAR T-cells.

### Functional Comparison of CD19-Specific CAR T-Cells

**Figure 3:**
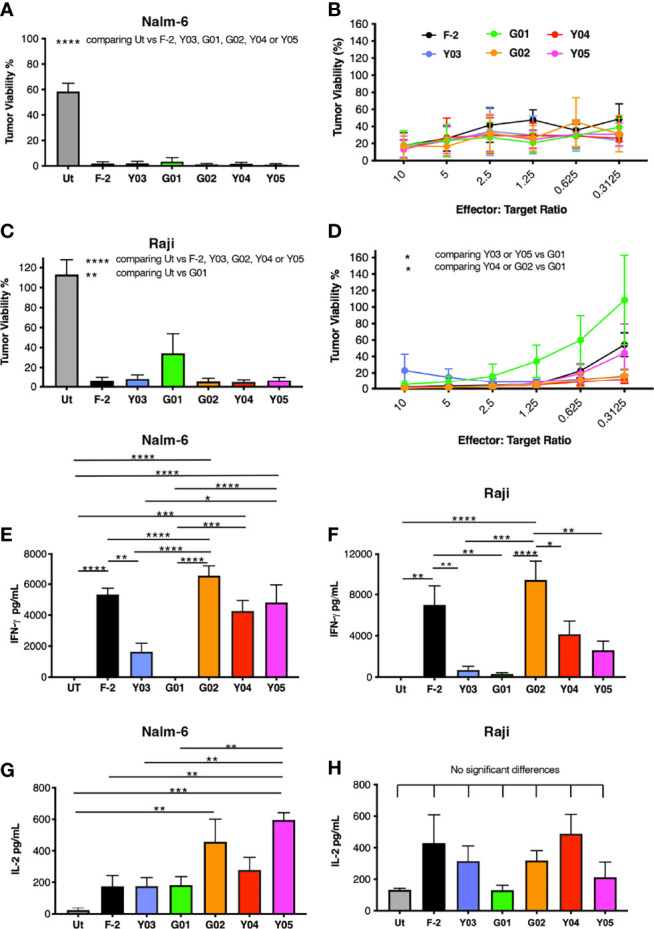
*In vitro* activation of scFv mutated CD19-specific CARs. **(A)** 2x10^4^ of the indicated CAR T-cell populations were co-cultivated with NALM-6 leukemic cells [1:1 ratio – **(A)**; indicated effector to target cell ratio - **(B)**] or Raji cells [1:1 ratio – **(C)**; indicated effector to target cell ratio - **(D)**]. After 72h, residual viability of tumor cells was determined by luciferase assay (mean ± SEM of n=3 independent replicates). Supernatants were collected 24h after co-cultivations described in **(A, C)** were established. These were analyzed for IFN-γ [Nalm-6, **(E)**; Raji, **(F)**] or IL-2 [Nalm-6, **(G)**; Raji, **(H)**] by ELISA (mean ± SEM of n=6 independent replicates). Statistical analysis was performed by one-way ANOVA **(A, C, E–H)** or two-way ANOVA **(B, D)** followed by Tukey’s multiple comparison test. Ut. - untransduced T-cells. ****p < 0.0001; ***p ≤ 0.001; **p ≤ 0.01; *p ≤ 0.05.

Next, we compared the *in vitro* anti-tumor activity of this panel of CD19-specific CARs. All five scFv modified CARs mediated the cytotoxic destruction of the malignant B-cell lines, Nalm-6 (**Figures 3A, B**) and Raji (**Figures 3C, D**). Expression of CD19 on these tumor cells in addition to CD19-engineered LO68 cells is shown in **Figure S2**. This was accompanied by production of IFN-γ (Nalm-6, **Figure 3E**; Raji, **Figure 3F**) and IL-2 (Nalm-6, **Figure 3G**; Raji, **Figure 3H**). Impaired cytotoxicity against Raji cells and reduced cytokine production was noted for T-cells that expressed the G01 CAR, in keeping with its very weak CD19 binding activity and poor avidity for LO68-CD19 tumor cells. Activated T-cells that expressed the Y03 CAR also produced lower levels of IFN-γ, in accordance with the reduced ability of this CAR to bind CD19 and lowered avidity of these cells for LO68-CD19 cells. However, the G02 CAR demonstrated a trend towards enhanced cytokine release when compared to the parental F-2 CAR, despite low binding of CD19-Fc. Notably, G02 CAR T-cells retained an intermediate avidity for CD19-expressing LO68 tumor cells, a property that was shared with Y05 CAR T-cells.

### Comparison of *In Vitro* Function of CD19-Specific CARs and Parallel CARs

**Figure 4:**
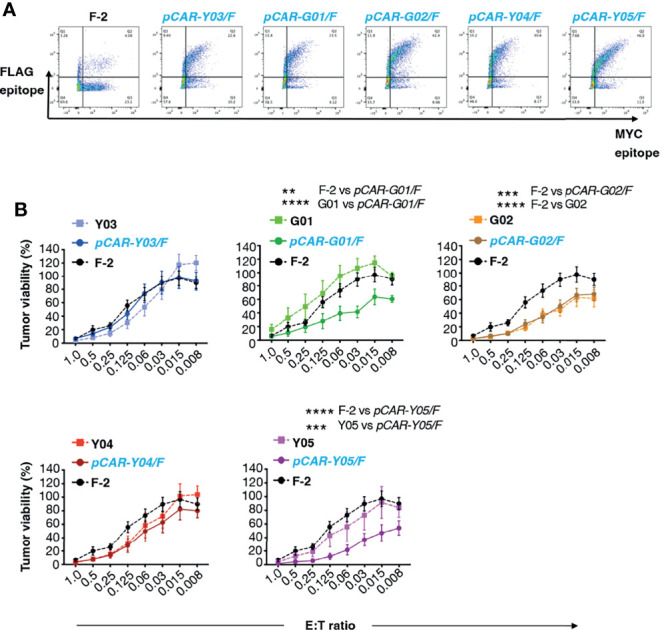
Evaluation of cytolytic activity of pCARs targeted against CD19. **(A)** Human T-cells were engineered by retroviral transduction to express the indicated pCARs, or F-2 CAR as control. Permeabilized T-cells were incubated with antibodies directed against embedded MYC (CAR) and FLAG (CCR) epitope tags and then analyzed by flow cytometry. Data are representative of three independent replicate experiments. **(B)** CAR or pCAR T-cells were co-cultivated with ffLuc/RFP^+^ Nalm-6 leukemia cells at the indicated E:T ratio. Target viability was quantified after 72h (mean ± SEM, n=7 replicates from 5 donors). Statistical analysis was performed using two-way ANOVA. **p < 0.01; ***p < 0.001; ****p < 0.0001.

We have recently demonstrated superior anti-tumor function of pCAR T-cells, in which a CD28-containing 2G CAR is co-expressed with a 4-1BB-containing CCR (10). To test the applicability of the pCAR platform to targeting of CD19, we co-expressed each of the mutant scFv-based 2G CARs described above with a 4-1BB CCR that contains an unmodified FMC63 scFv. We selected this arrangement since we have found that excessive affinity (e.g. low picomolar k_d_) compromises CAR, but not CCR function, in the context of a pCAR (unpublished data). The FMC63 scFv has high affinity for CD19 whereas all mutated scFv derivatives mediate similar or lower avidity for CD19-expressing target cells. Parallel CARs are named as *pCAR-X/Y* where X and Y respectively are abbreviations for the targeting moiety used in the CAR (i.e. mutated FMC63 scFv such as G02) and CCR (i.e. unmodified FMC63 scFv, abbreviated as F) (**Figure 1C**). Cell surface 1:1 co-expression of CAR and CCR components was demonstrated by flow cytometry (**Figure 4A**).

**Figure 5:**
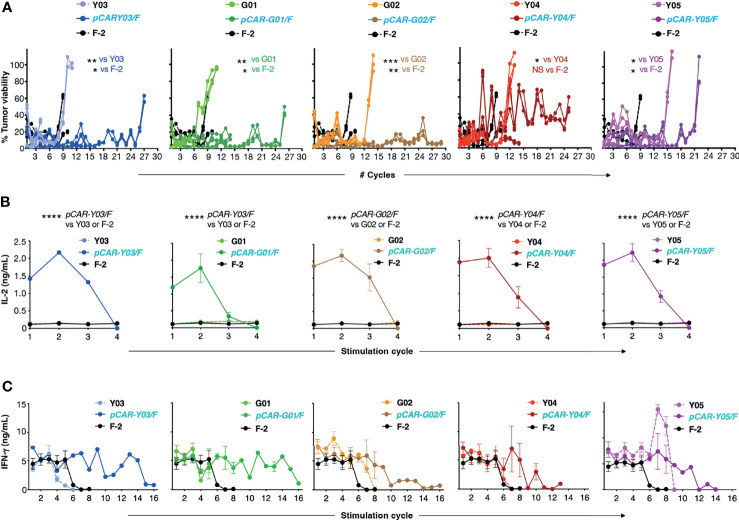
Re-stimulation of CD19-targeted CAR and pCAR T-cells. **(A)** T-cells were engineered to express the indicated CARs or pCARs. 1x10^5^ of the indicated transduced CAR or pCAR T-cells were co-cultivated in triplicate in a 1mL volume with an equal number of LO68-CD19^+^ tumor cells. After 72h, T-cells were transferred to a fresh monolayer of CD19^+^ LO68 cells and viability of the original tumor monolayer was determined by MTT assay. Plots indicate tumor cell viability at each stimulation cycle. Cultures were terminated when tumor cell killing was <60% or if T-cells could not be recovered. Statistical analysis was performed using an unpaired Student’s *t* test comparing the number of stimulation cycles achieved by each pCAR compared to the indicated CAR. Supernatant was collected 24h after the initiation of each stimulation cycle and was analyzed by ELISA for IL-2 [mean +/- SEM, n=2; **(B)**] or IFN-γ [mean +/- SEM, n=6; **(C)**] (mean +/- SEM of duplicate independent cultures). Statistical analysis was performed using two-way ANOVA. ****p= < 0.0001; ***p= ≤ 0.001; **p= ≤ 0.01; *p < 0.05.

Next, we compared the anti-tumor activity of these CAR and pCAR T-cells *in vitro*. In cytotoxicity assays performed at a range of low effector to target ratios, the G02 CAR demonstrated significantly enhanced killing activity against Nalm-6 leukemic cells compared to the parental F-2 CAR (**Figure 4B**). Cytolytic activity of all pCAR T-cell populations was similar or superior compared to the corresponding mutant scFv-based 2G CAR, or the F-2 CAR (**Figure 4B**). We have previously shown that a docking effect of the CCR can potentiate cytolytic activity of some, but not all pCARs, when compared to the parental CAR (10). Analysis of immune synapse formation by these T-cell populations could help to uncover mechanisms that may explain this variable response of pCAR T-cells. Parallel CAR T-cells strongly outperformed those that expressed the corresponding mutant scFv-based CAR or F-2 in tumor re-stimulation assays, maintaining cytolytic activity over a significantly greater number of stimulation cycles in every case (**Figure 5A**). Strikingly, pCAR T-cells produced significantly more IL-2 over early tumor re-stimulation cycles, unlike CAR T-cells in which specificity was conferred by the same mutated scFv or F-2 (**Figure 5B**). A trend towards more sustained IFN-γ production upon iterative tumor re-stimulation was also observed for pCAR T-cells, compared to T-cells that expressed the corresponding CAR or F-2 (**Figure 5C**).

**Figure 6:**
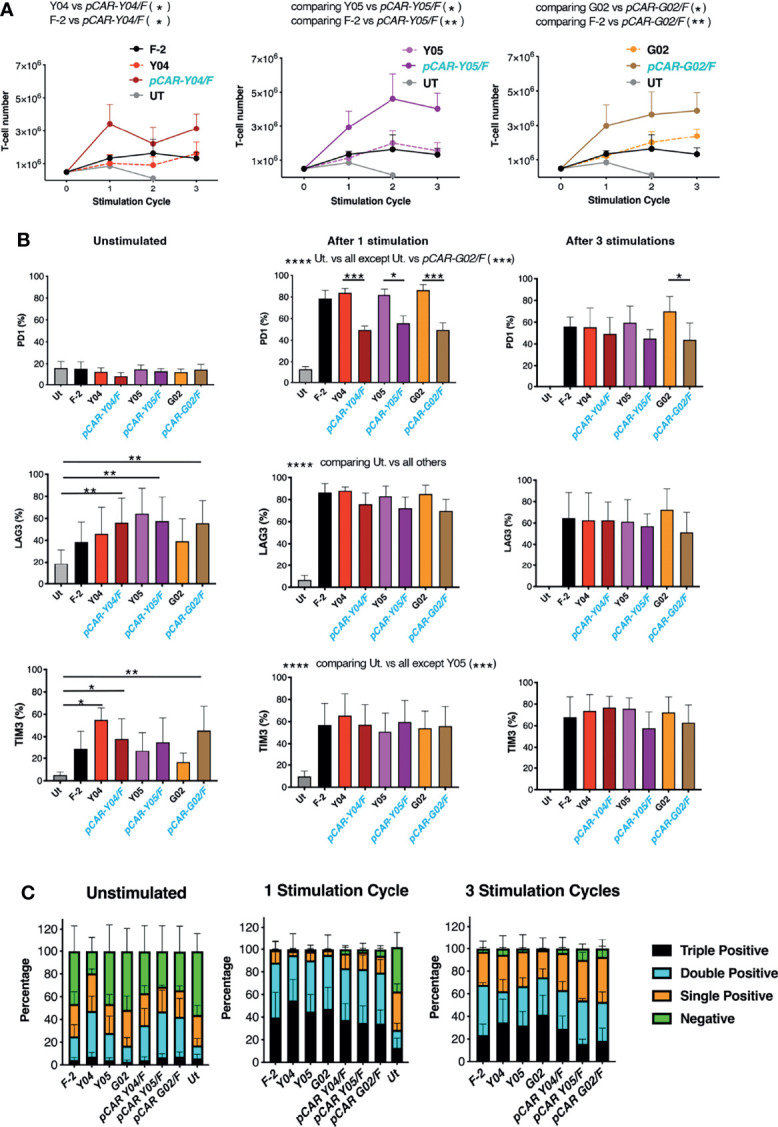
Expansion of tumor re-stimulated pCAR T-cells is accompanied by reduced exhaustion marker expression. **(A)** T-cells were engineered to express the indicated CARs or pCARs. 5x10^5^ of the indicated transduced CAR or pCAR T-cells were co-cultivated with 2.5x10^5^ LO68-CD19 tumor cells. After 72h, T-cells were harvested, counted and transferred to a new tumor monolayer. Data show T-cell number at the time of each stimulation cycle (mean ± SEM, n=3-4). Statistical analysis was performed using an unpaired Student *t*-test, comparing cell number between the indicated groups over stimulation cycles 1-3. *p < 0.05; **p < 0.01. **(B)** T-cells described in A that were unstimulated or 72 hours after the indicated number of stimulation (stim.) cycles were analyzed by flow cytometry for expression of PD1, LAG3 and TIM3. Statistical analysis was performed using two-way ANOVA with Tukey’s multiple comparisons. *p < 0.05; **p < 0.01; ***p < 0.001; ****p < 0.0001. **(C)** Bar graph of exhaustion marker data shown in **(B)** Segments indicate cells that were negative or were triple, double or single positive for the exhaustion markers PD1, LAG3 and/or TIM3 (mean ± SEM).

Given these findings, the best performing CARs and pCARs (Y04, Y05, G02 and derived pCARs) were advanced for further study, making comparison with the F-2 2G CAR and untransduced T-cells. Upon iterative tumor cell re-stimulation in the absence of exogenous cytokine support, all three pCARs mediated significantly greater T-cell expansion when compared to CAR alone or F-2 (**Figure 6A**). Analysis of exhaustion marker expression demonstrated that PD1 was highly upregulated when either CAR or pCAR T-cells were stimulated on LO68-CD19 tumor monolayers (**Figure 6B**). However, PD1 levels after one stimulation cycle were significantly lower on pCAR T-cells compared to the matched CAR and this also remained the case for *pCAR-G02/F* pCAR T-cells after the third stimulation cycle (**Figure 6B**). While no differences between CAR and matched pCAR T-cells were noted for either LAG3 or TIM3 expression (**Figure 6B**), repeatedly stimulated *pCAR-Y05/F* and *pCAR-G02/F* T-cell cultures demonstrated a trend towards reduced numbers of cells that co-expressed all three exhaustion markers (triple pos.; **Figure 6C**).

### *In Vivo* Comparison of CD19-Specific CAR and pCAR T-Cells

**Figure 7:**
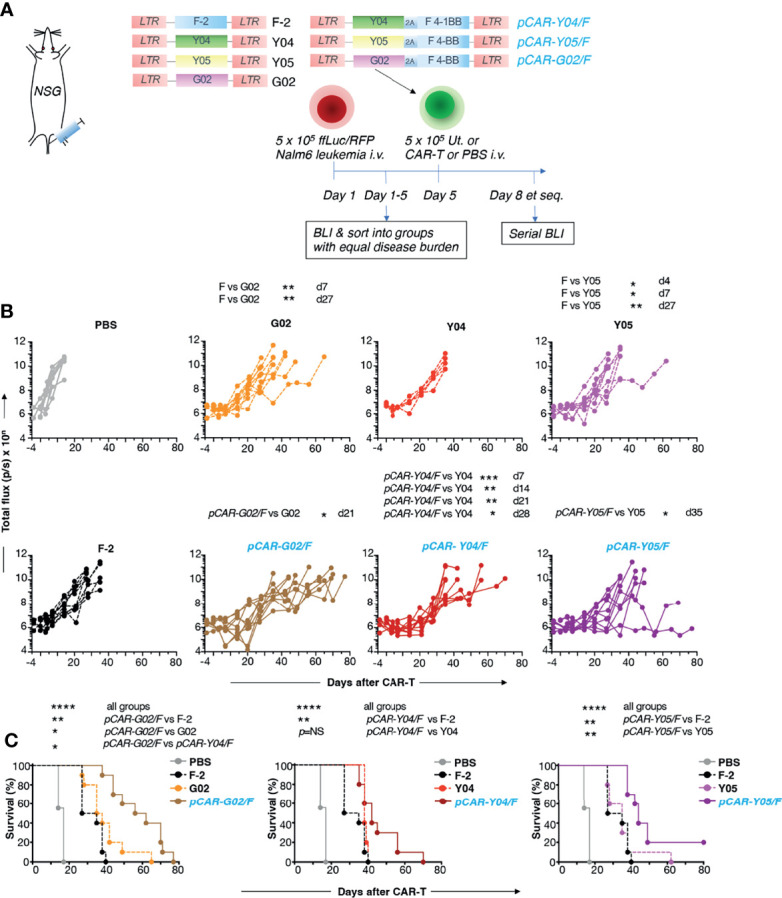
*In vivo* evaluation of pCARs targeted against CD19. **(A)** NSG mice (n=5-10 per group) were inoculated i.v. with 5 x 10^5^ ffLuc/RFP^+^ Nalm-6 cells. On day 5, mice with established leukemia were treated i.v. with 5 x 10^5^ CAR or pCAR T-cells or PBS. **(B)** Tumor burden in individual mice following treatment was monitored by BLI. Data are pooled from 2 donors. Statistical analysis was performed using an unpaired Student *t* test comparing the indicated CARs/pCARs. **(C)** Survival curves of mice. Statistical analysis was performed using a Log-rank (Mantel-Cox) test, comparing the indicated groups. *p < 0.05; **p < 0.01; ***p < 0.001; ****p < 0.0001.

*In vivo* anti-tumor activity of the advanced panel of CAR and pCAR T-cells was compared with the commonly used CD19^+^ Nalm-6 xenograft model of B-cell acute lymphoblastic leukemia (B-ALL), using BLI to monitor disease status. The experimental scheme used in the treatment of mice with an established disease burden is indicated in **Figure 7A**. Pooled results of two independent experiments are shown. CAR and pCAR T-cell transduction efficiency was determined 24 hours prior to i.v. infusion by flow cytometry and the transduction efficiency was normalized across groups by the addition of untransduced T-cells. Following CAR/pCAR T-cell treatment, all animals were monitored by BLI weekly. At the modest CAR/pCAR T-cell dose employed (5 x 10^5^ cells), F-2 CAR T-cells caused a delay in leukemic progression compared to PBS, while the two intermediate avidity 2G CAR derivatives (G02 and Y05) achieved a further significant improvement in disease control (**Figure 7B**). Importantly, all pCARs achieved significantly enhanced disease control (**Figure 7B**) and extended survival (**Figure 7C**) when compared to F-2 or their CAR of origin. Survival was greatest when pCARs contained an intermediate avidity CAR (e.g. G02 or Y05), rather than a high avidity CAR (e.g. Y04), reaching significance in the comparison between *pCAR-G02/F* and *pCAR-Y04/F*. Clinical evidence of CAR T-cell-induced toxicity was not apparent in any treatment group, nor was non-tumor-related weight loss observed following treatment (**Figure S3**). It should be noted however that cytokine release syndrome cannot be accurately modeled in NSG mice owing to the lack of fully functional macrophages in these animals (25). Parallel CAR T-cells demonstrated enhanced *in vivo* persistence compared to control F-2 2G CAR T-cells (**Figure S4**). Together, these data demonstrate that the pCAR platform enables the delivery of superior anti-tumor activity using scFv targeting moieties and across a broad range of relative CAR/CCR binding strengths.

## Discussion

While the clinical success of CD19-specific CAR T-cells provides tremendous encouragement, there remains a need to further improve remission rates and durability of disease control. We hypothesized that this may be achieved using pCAR T-cells that harness dual co-stimulation by CD28 and 4-1BB. Parallel CARs consist of the stoichiometric co-expression of a CD28+CD3ζ 2G CAR with a 4-1BB containing CCR. Using a range of tumor models, we have previously demonstrated that pCAR T-cells outperform linear CARs that contain one or two co-stimulatory domains or alternative configurations that place one co-stimulatory domain away from the plasma membrane (10). Parallel CAR T-cells also undergo reduced apoptosis upon iterative tumor re-stimulation (10). Broadly similar results are obtained if co-stimulatory modules are switched between CAR and CCR (data not shown). In keeping with these advantageous properties of pCAR T-cells, administration of an anti-4-1BB antibody boosts anti-tumor activity of CD28-containing 2G CAR T-cells (26). Moreover, provision of either CD28 or 4-1BB co-stimulation to an activated T-cell *in cis* is markedly more potent than when either is provided *in trans* (27).

In this study, we have evaluated the suitability of the pCAR platform for the treatment of CD19-expressing malignancy with a view to identifying an optimal candidate for clinical advancement. The FMC63 scFv is most commonly used in CD19 CARs and has low nanomolar affinity for this antigen (K_a_ 2.3 x 10^-9^) (23). Some prior studies have indicated that high affinity CARs retain satisfactory anti-tumor activity, whereas lower affinity derivatives have superior capacity to discriminate between tumor cells that express high levels of target antigen and normal cells in which target antigen is found at lower levels (28–30). However, studies with TCRs have indicated that that there is an affinity ceiling above which increased binding strength adversely affects T-cell response (31–33). This phenomenon has also been reported for some high affinity CARs which retain and sequester target ligand efficiently, potentially preventing serial killing of tumor cells (34). Clinical evidence in support of this concept arises from a recent trial involving B-ALL. Patients were treated with CAR T-cells that had >40-fold reduction in affinity for CD19 compared to FMC63 CARs. The lower affinity CAR demonstrated superior performance in pre-clinical testing and maintained excellent efficacy, but without any severe toxicity when evaluated in man (35). For this reason, we undertook mutagenesis of the FMC63 scFv in order to generate a panel of derivates that encompassed a spectrum of CD19 binding strengths.

Commonly, CAR affinity is inferred from biophysical studies performed using a soluble form of the targeting moiety, such as an scFv-Fc fusion (35). However, this is a time-consuming undertaking which requires expression and purification of the panel of scFvs under study prior to undertaking binding studies, for example using surface plasmon resonance biosensors such as the BIAcore protein interaction platform. While this provides useful information regarding the scFv itself, this analysis does not consider the geography of chimeric antigen receptor or target antigen expression within the cell membrane, nor the influence of secondary binding interactions mediated by other pro-adhesive molecules. Accordingly, some studies of scFv affinity have proven poorly predictive of CAR function (36). To measure the global strength of interaction between our panel of CD19-specific CAR T-cells and a target cell that expresses this antigen, we undertook z-Movi avidity testing. These CAR T-cells demonstrated a spectrum of avidities with strongest interaction mediated by the original F-2 CAR and the Y04 mutant. The G01 and Y03 mutants had low avidity in agreement with CD19-binding studies. Intermediate avidity was observed with the Y05 and G02 mutants, despite the greater ability of the former to bind CD19-Fc. Notably, both of these CAR T-cells demonstrated significantly greater *in vivo* anti-tumor activity in the Nalm-6 leukemic xenograft model, when compared to the parental F-2 CAR. Conceptually, CAR T-cells with intermediate avidity may have a superior ability to dock transiently on target cells, enabling serial tumor cell killing (37) and a reduction in deleterious effects of over-activation, such as exhaustion and activation-induced cell death (38). Despite its poor ability to bind CD19, the superior target cell avidity and anti-tumor activity of the G02 CAR suggests that it can recruit additional adhesive mechanisms to achieve productive target cell engagement, perhaps *via* enhanced immune synapse formation.

We proceeded to generate pCAR derivatives of these CARs by co-expression of a CCR in which an unmodified FMC63 scFv was coupled *via* a CD8α spacer and transmembrane domain to a 4-1BB endodomain. As observed with previously described pCARs (10), all CD19-specific pCAR T-cells achieved markedly enhanced tumor re-stimulation activity, maintained capacity to produce IL-2 over repeated stimulation and reduced expression of exhaustion markers, most notably PD1. Enhanced cytokine production by pCAR compared to second-generation CAR T-cells is consistent with the effects of dual co-stimulation *via* CD28 and 4-1BB, as described previously (39). Conversion to a pCAR system also enhanced the cytolytic activity of G01 CAR T-cells, despite poor CD19 binding and low avidity of these T-cells for CD19-expressing targets. This suggests that the CCR may contribute to target cell docking under some circumstances, as described for other pCARs (10). In keeping with this, recently published data indicate that co-expression of a CCR together with a CAR leads to increased functional avidity and enhanced sensitivity to detect tumor cells that express low levels of CAR target antigen (40). When administered at a low dose to mice with an established Nalm-6 leukemic burden, all pCAR T-cells significantly outperformed their CAR counterparts, or the parental F-2 CAR, in which an unmodified FMC63 scFv conferred CD19 specificity. Accordingly, disease progression was delayed and survival enhanced, without significant toxicity. In particular, the *pCAR-G02/F* pCAR achieved the greatest survival advantage, suggesting that intermediate CAR avidity may also be a favorable attribute of pCAR T-cells, at least under conditions of high CD19 expression. To further characterize this platform, additional studies that compare anti-tumor activity of all configurations of high and/or low avidity targeting moieties in the CAR and CCR would be useful. Additional testing of T-cells from a larger donor panel, including patients with B-cell malignancy, and testing on target cells with different antigen densities would provide further useful confirmatory information. Taken together, these data support the clinical evaluation of CD19 pCAR immunotherapy in patients with relapsed refractory B-cell malignancy.

## Data Availability Statement

Publicly available datasets were analyzed in this study. This data can be found here: https://www.ncbi.nlm.nih.gov/nuccore/, HM852952.1.

## Ethics Statement

The studies involving human participants were reviewed and approved by London Bridge Research Ethics Committee and Research Ethics Committee 3, West of Scotland. The patients/participants provided their written informed consent to participate in this study. The animal study was reviewed and approved by King’s College London Animal Welfare and Ethical Review Body.

## Author Contributions

Conceptualization, JM. Experimental work, LH, KD, DL-Y, AA, and DD. Methodology and data analysis, all authors. Project Oversight AC, RD, and JM. Writing JM. Review and editing, all authors. All authors contributed to the article and approved the submitted version.

## Funding

This work was supported by Leucid Bio, the British Lung Foundation (MESOUK18-2 INT 1), the Medical Research Council (MR/R001936/1), the Experimental Cancer Medicine Centre at King’s College London, the King’s Health Partners/King’s College London Cancer Research UK Cancer Centre and by the National Institute for Health Research (NIHR) Biomedical Research Centre based at Guy’s and St Thomas’ NHS Foundation Trust and King’s College London (IS-BRC-1215-20006).

## Author Disclaimer

The views expressed are those of the authors and not necessarily those of the NHS, the NIHR or the Department of Health.

## Conflict of Interest

JM is CSO, scientific founder and shareholder of Leucid Bio, is a member of the scientific advisory board of Arovella Pharmaceutics Ltd and has undertaken consultancy work for Bristol-Meyers-Squibb, Juno, Celgene, Ellipses Pharma and Biotest. LH undertook a PhD studentship funded by Leucid Bio. DD and DL-Y have acted as consultants for Leucid Bio and DD has been employed by Leucid Bio following completion of this work. JM and LH are co-inventors on patent filings in relation to pCAR technology. KD and AC are employees of Lumicks.

The remaining authors declare that the research was conducted in the absence of any commercial or financial relationships that could be construed as a potential conflict of interest.

## Publisher’s Note

All claims expressed in this article are solely those of the authors and do not necessarily represent those of their affiliated organizations, or those of the publisher, the editors and the reviewers. Any product that may be evaluated in this article, or claim that may be made by its manufacturer, is not guaranteed or endorsed by the publisher.

## Footnotes

1 http://www.abysis.org/abysis/, last accessed Nov 5^th^, 2021.
