# Supplementary material for: Engineering of an Avidity-Optimized CD19-Specific Parallel Chimeric Antigen Receptor That Delivers Dual CD28 and 4-1BB Co-Stimulation
Source: Front Immunol. 2022 Feb 9;13:836549. doi: 10.3389/fimmu.2022.836549 (PMC8863855; doi:10.3389/fimmu.2022.836549)
Supplement: Supplementary file 1 [file DataSheet_1.pdf]

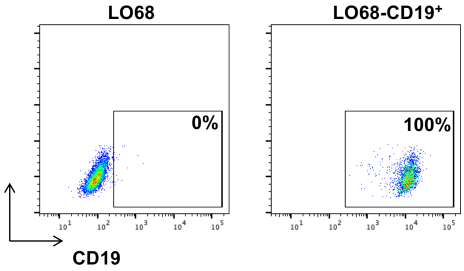

Fig. S1. Retrovirus transduced LO68 cells engineered to express CD19. The mesothelioma line LO68 was transduced with an SFG retroviral vector that encodes for human CD19. Antigen expression was detected by flow cytometry after incubation with a FITC-conjugated anti-CD19 antibody.

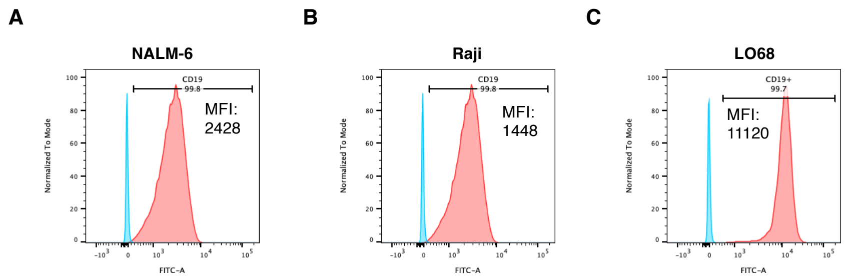

Fig. S2. Expression of CD19 on tumor cell lines. Mean fluorescence intensity of CD19 expression (red) is shown for Nalm-6 (**A**), Raji (**B**) and LO68 cells engineered to express CD19 (**C**), making comparison with isotype control (blue).

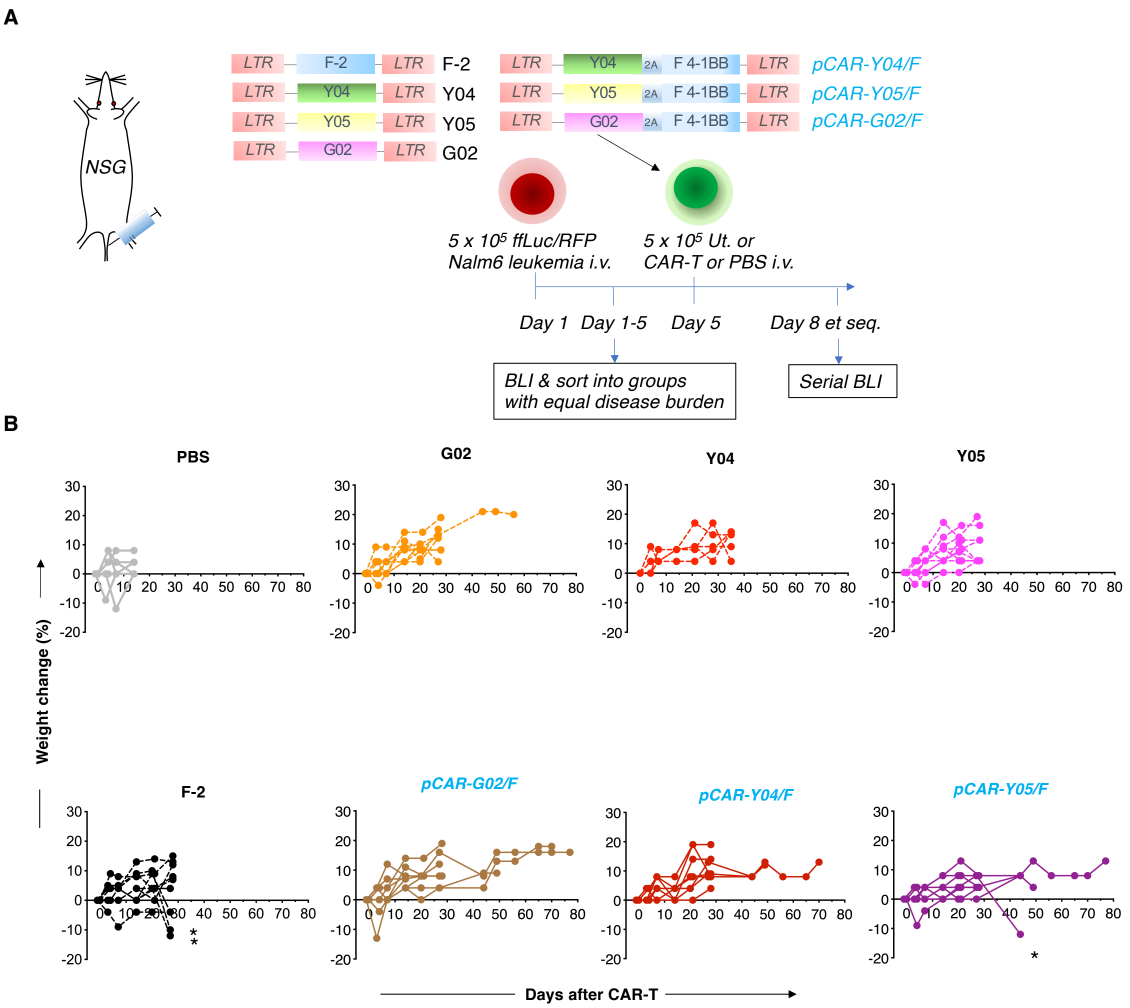

Fig. S3. *In vivo* evaluation of pCARs targeted against CD19. NSG mice (n=5-10 per group) were inoculated i.v. with 5 x 10^5 ffLuc/RFP^+ Nalm6 cells. On day 5, mice with established leukemia were treated i.v. with 5 x 10^5 CAR or pCAR T-cells or PBS. Serial weight change of mice is shown. Animals with significant weight loss attributable to increasing tumor burden are indicated (*).

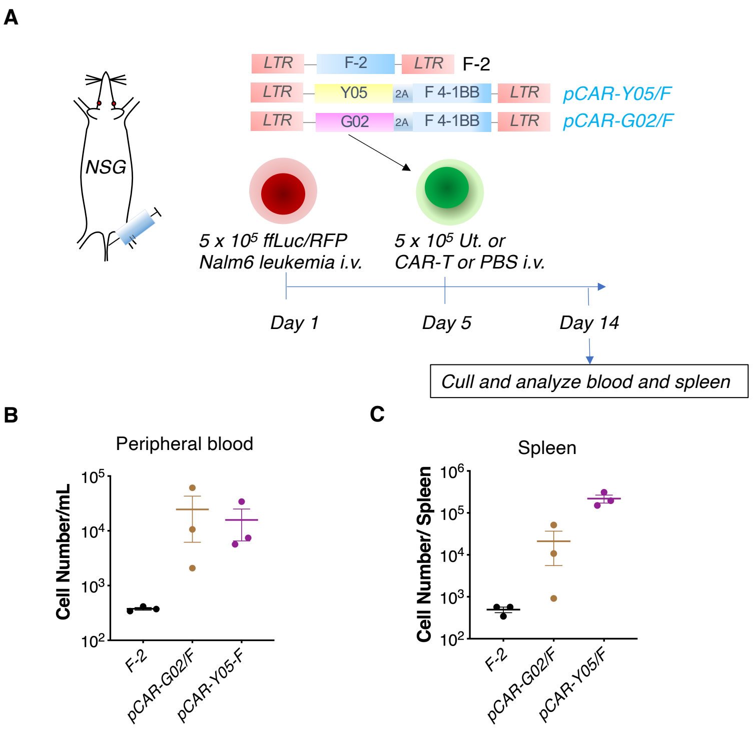

Fig. S4. *In vivo* evaluation of pCARs targeted against CD19. **(A)** NSG mice (n=3 per group) were inoculated i.v. with 5 x 10^5 ffLuc/RFP$^+$ Nalm6 cells. On day 5, mice with established leukemia were treated i.v. with 5 x 10^5 CAR or pCAR T-cells or PBS. Animals were culled on day 14 and CAR T-cell numbers evaluated in peripheral blood (**B**) and spleen (**C**) by flow cytometry (mean $\pm$ SEM).
